# Quantifying the effects of temperature on mosquito and parasite traits that determine the transmission potential of human malaria

**DOI:** 10.1371/journal.pbio.2003489

**Published:** 2017-10-16

**Authors:** Lillian L. M. Shapiro, Shelley A. Whitehead, Matthew B. Thomas

**Affiliations:** The Pennsylvania State University Department of Entomology and Center for Infectious Disease Dynamics, University Park, Pennsylvania, United States of America; Stanford University, United States of America

## Abstract

Malaria transmission is known to be strongly impacted by temperature. The current understanding of how temperature affects mosquito and parasite life history traits derives from a limited number of empirical studies. These studies, some dating back to the early part of last century, are often poorly controlled, have limited replication, explore a narrow range of temperatures, and use a mixture of parasite and mosquito species. Here, we use a single pairing of the Asian mosquito vector, *An*. *stephensi* and the human malaria parasite, *P*. *falciparum* to conduct a comprehensive evaluation of the thermal performance curves of a range of mosquito and parasite traits relevant to transmission. We show that biting rate, adult mortality rate, parasite development rate, and vector competence are temperature sensitive. Importantly, we find qualitative and quantitative differences to the assumed temperature-dependent relationships. To explore the overall implications of temperature for transmission, we first use a standard model of relative vectorial capacity. This approach suggests a temperature optimum for transmission of 29°C, with minimum and maximum temperatures of 12°C and 38°C, respectively. However, the robustness of the vectorial capacity approach is challenged by the fact that the empirical data violate several of the model’s simplifying assumptions. Accordingly, we present an alternative model of relative force of infection that better captures the observed biology of the vector–parasite interaction. This model suggests a temperature optimum for transmission of 26°C, with a minimum and maximum of 17°C and 35°C, respectively. The differences between the models lead to potentially divergent predictions for the potential impacts of current and future climate change on malaria transmission. The study provides a framework for more detailed, system-specific studies that are essential to develop an improved understanding on the effects of temperature on malaria transmission.

## Author summary

Many of the mosquito and parasite life history traits that combine to influence the transmission intensity of malaria (e.g., adult mosquito longevity, biting rate, the developmental period of the parasite within the mosquito, and the proportion of mosquitoes that become infectious) are strongly temperature sensitive. Yet, in spite of decades of research, the precise relationships between individual traits and temperature remain poorly characterized. As a consequence, the majority of studies exploring the influence of local environmental conditions, or prospective impacts of climate change, draw on a combination of studies that utilize different experimental methods and a range of mosquito and parasite species. Here, we use the Indian malaria mosquito, *Anopheles stephensi*, and the human malaria parasite, *Plasmodium falciparum*, to thoroughly characterize the influence of temperature on key transmission-related traits. The results reveal a number of novel insights and challenge some longstanding assumptions regarding the nature of mosquito and parasite thermal responses. This study provides an experimental blueprint for further system-specific studies necessary to more fully understand the implications of changing temperatures on malaria transmission.

## Introduction

Numerous studies show the transmission of malaria to be strongly influenced by environmental temperature [1–10]. This has led to a large body of both theoretical and empirical research which explores the possible effects of temperature on the dynamics and distribution of malaria both in the present day [6, 11–16] and under scenarios of future climate change [4, 7, 8, 17–23]. In spite of the accepted importance of temperature, the thermal sensitivity of individual mosquito and parasite traits that combine directly or indirectly to determine transmission intensity (i.e., adult longevity, biting rate, fecundity, generation time, vector competence, and parasite extrinsic incubation period [EIP]) remains surprisingly poorly characterized. For example, a recent study that explored the influence of temperature on transmission rate of *P*. *falciparum* in Africa utilized data from a Latin American malaria vector for biting rate, a North American vector infected with *P*. *vivax* for vector competence, a mixture of 6 malaria vector species from Asia, Africa, and North America for parasite development rate, and even a nonmalaria vector (*Aedes albopictus*) for fecundity [4]. Many other studies rely on similar data [6, 8, 13, 17, 24–26]. The necessity to combine insights from such disparate systems highlights the paucity of data.

Similarly, the iconic degree-day model developed in the 1960s to define the EIP (also called the period of sporogony) of *P*. *falciparum* inside the mosquito vector [27, 28] has been applied in a multitude of studies over the years. Yet, it is rarely acknowledged that this relationship derives from a limited number of experiments conducted in the 1930s and 1940s by using Russian populations of native Mediterranean mosquitoes (*An*. *maculipennis*). Furthermore, many other historical experiments that explore temperature-sensitive parasite development rate are very poorly replicated (data points sometimes based on single mosquitoes), contain little or no information about the sources of infectious blood or blood infection levels, and explore a limited temperature range [29–33]. Whether these data are sufficient to describe parasite development rate in every malaria transmission system seems extremely unlikely, yet that is the prevailing assumption.

Here, we present a detailed investigation of how temperature affects key mosquito and parasite traits for a single species pairing of *An*. *stephensi* and *P*. *falciparum* across a range of temperatures relevant to malaria transmission. Specifically, we measured adult mosquito mortality rate, the duration of the EIP (the time for parasites to reach their infectious stage), vector competence (the maximum prevalence of infectious mosquitoes), and biting rate across several temperatures from 21°C to 34°C. We then use these data to generate temperature-dependent models of relative vectorial capacity (rVC) and relative force of infection. Our data and the contrasting models highlight the need to improve empirical understanding of the effects of temperature on malaria transmission in addition to providing an experimental framework for conducting future species-specific research across a range of vector-parasite pairings.

## Results

Our principal experiment involved feeding replicate cups of approximately 150, 3 to 5 day-old adult female *An*. *stephensi* mosquitoes on human blood infected with *P*. *falciparum*. We then transferred these mosquitoes to temperature-controlled incubators set to 21, 24, 27, 30, 32, and 34°C (our pilot experiment also contained groups at 16°C and 18°C, but no sporogony had occurred through day 26 postinfection, so those groups were excluded from further experiments; see S1 Table). Mosquitoes were monitored to assess daily mortality, and subsamples were removed for dissections at set intervals (see Materials and methods) to track the time it took for parasites to invade the salivary glands, and hence, the distribution of time over which mosquitoes became infectious. The experiment was repeated over 2 independent experimental blocks.

### Mosquito mortality

**Fig 1:**
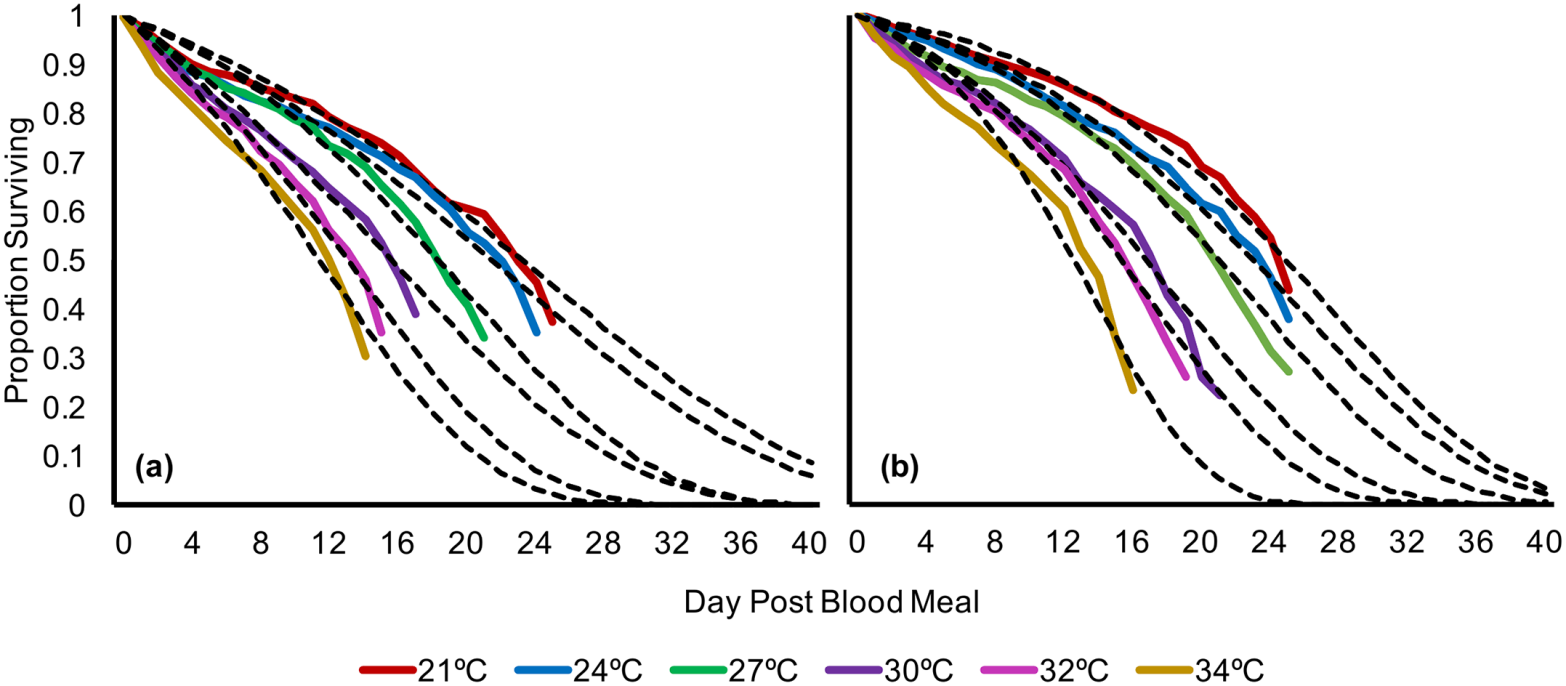
Gompertz model predictions for each temperature and block combination overlaid on corresponding raw (Kaplan-Meier) survival data; (A) experimental block 1; (B) experimental block 2. Raw data and numerical values can be accessed at http://dx.doi.org/10.5061/dryad.74389 [34].

We found that mosquito mortality rate was significantly different across temperature (log-rank test, χ^2^ = 533, df = 5, *p* < 0.001) and across temperature and block (log-rank test, χ^2^ = 569, df = 11, *p* < 0.001). Upon pairwise log-rank comparison analysis, we found several instances of significant effects of block x temperature interactions (S2–S6 Tables). These interactions are likely due to decreased initial mortality in the second block, which allowed for increased sampling time, especially across warmer temperatures (30°C to 34°C). Nonetheless, mortality tended to increase with temperature across both experimental blocks (Fig 1).

Due to logistic constraints in generating large numbers of infected mosquitoes, we tracked both mortality and sporogony within the same mosquito samples and, thus, do not have survival data that encompass the entire mortality curve without censored cases. We used parametric survival analysis to characterize survival of each temperature group (S7 and S8 Tables). We fit several survival distributions (Gompertz, Weibull, exponential, log-logistic, and log-normal) to our data and compared the fits using the Aikake information criterion (AIC) (S7 and S8 Tables). These models have been used to describe survival curves of a diversity of insects in laboratory and field settings [35–42]. Our cumulative survival data were best described by a Gompertz function, in which the mortality hazard increases exponentially with age:

$$f\left(x\right)=\alpha e^{\beta x}$$

where *x* is a given age, *α* is the initial mortality rate, and *β* is the constant exponential mortality increase with age [26, 35, 43, 44]. Because of significant interactions driven by block differences, we built a Gompertz function that describes each temperature x block combination separately (Fig 1, Table 1). Overall, block 2 exhibited higher median survival times, along with lower initial mortality rate (α) values than block 1, regardless of the age-dependent exponential increase in mortality (β).

**Table 1:** Initial mortality rate (α), age-dependent exponential constant (β) and median survival time for each temperature and experimental block.

Temperature	Block	Initial Mortality Rate (α)	Age-dependent exponential constant (β)	Median Survival Time, Days (95% CI)
21°C^ab^	1	0.0129	0.0645	**23.2** (21.4–25.3)
21°C^c^	2	0.0063	0.1001	**24.9** (23.2–26.8)
24°C^d^	1	0.015	0.0642	**21.5** (19.9–23.2)
24°C^a^	2	0.0087	0.0929	**22.9** (21.5–24.6)
27°C^e^	1	0.0144	0.0928	**18.3** (17.1–19.7)
27°C^bd^	2	0.0103	0.0995	**20.5** (19.3–21.7)
30°C^f^	1	0.0236	0.0746	**15.6** (14.3–17.0)
30°C^e^	2	0.0152	0.1040	**16.8** (15.7–18.0)
32°C^g^	1	0.0257	0.1006	**13.1** (12.0–14.3)
32°C^f^	2	0.0161	0.1170	**15.4** (14.5–16.3)
34°C^h^	1	0.0315	0.1044	**11.4** (10.6–12.3)
34°C^g^	2	0.0172	0.1588	**12.6** (12.0–13.2)

Initial mortality rate (α) and age-dependent exponential mortality constant (β) of Gompertz functions calculated for each temperature and block combination along with calculated median survival time (bold) with 95% confidence intervals (in parentheses). Values represent calculated rates and survival times for all replicate cups pooled together per block. Superscripts indicate groups that are not significantly different (*p* > 0.05) upon post-hoc log-rank comparisons between each combination of block and temperature (for detailed information, see S6 Table).

### Parasite infection

**Fig 2:**
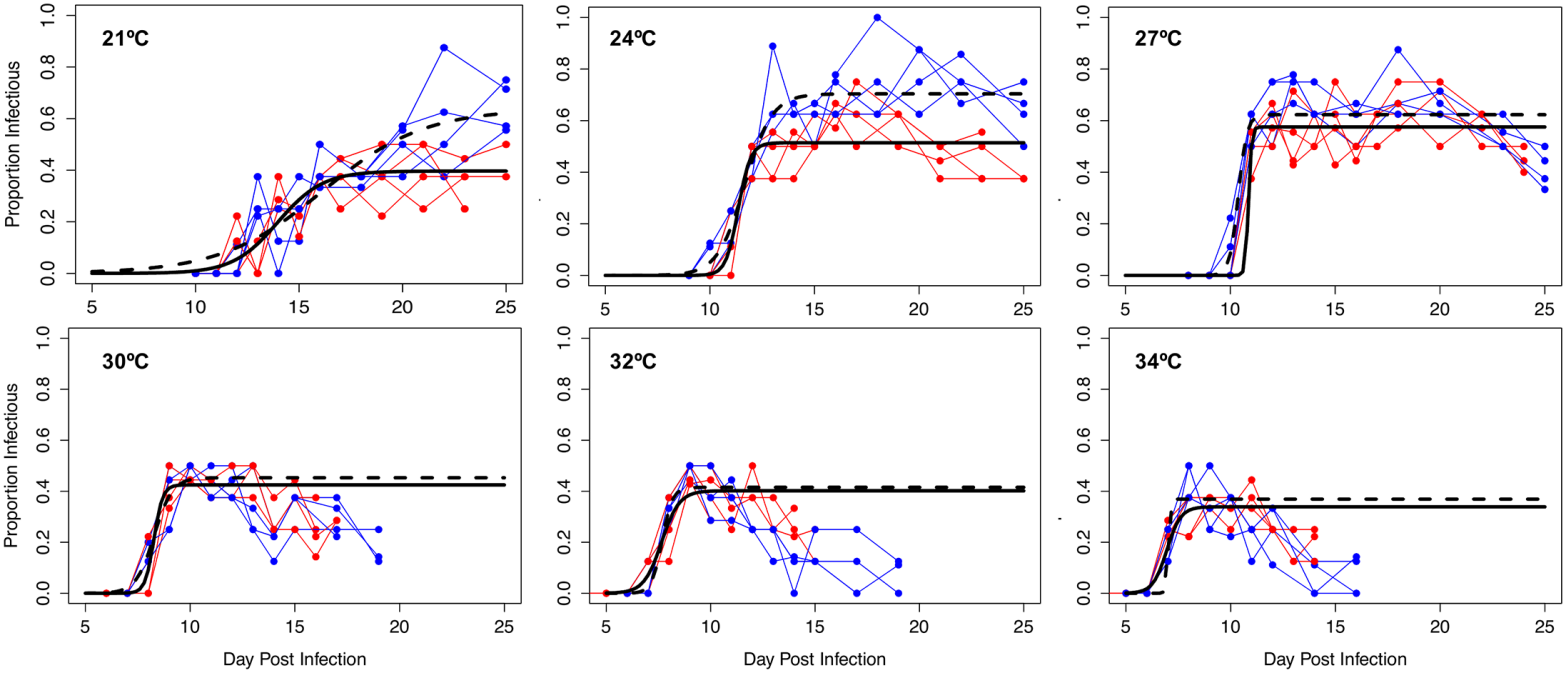
Dynamics of infectiousness over time for each temperature and block combination. Sporogony represented by the change in proportion of infectious mosquitoes over time. Blue points with connecting lines represent dynamics for each experimental cup in block 1; red points with connecting lines represent cup dynamics for block 2. The logistic regression model for block 1 is depicted by the solid black line, whereas the model for block 2 is the dashed black line. Raw data and numerical values can be accessed at http://dx.doi.org/10.5061/dryad.74389 [34].

Dissection of mosquitoes revealed an increase in the prevalence of sporozoite-infected mosquitoes over time in all temperatures (Fig 2). To describe this pattern and enable comparisons of EIP between temperatures, we fit a basic logistic curve to the data for each temperature in both experimental blocks:

$$g_{x}=\frac{g}{1+e^{-k\left(x-t\right)}}$$

where at any given day *x*, the proportion of infectious mosquitoes is dependent on *g* (the asymptote), which is the maximum sporozoite prevalence and provides a measure of vector competence, *k* (a rate constant), which describes the instantaneous change in proportion of infectious mosquitoes through time, and *t* (the inflection point), the time at which 50% of maximum proportion infectious is reached [1,45,46].

At 21°C, 24°C, and 27°C, sporogony was well described by the logistic function applied to the full data series. However, at 30°C, 32°C, and 34°C, the raw data showed an initial increase in prevalence of infectious mosquitoes followed by a decline, which was not accurately described by the logistic function alone (Fig 2). For subsequent modeling analysis (see section on Transmission potential below), we truncate the logistic curves at the day of the peak proportion of infectious mosquitoes and fit exponential curves to characterize the decline in prevalence (calculated by using nonlinear least squares regression in R; S9 Table). This truncation does not affect the calculation of the maximum proportion infectious, the rate constant, nor the inflection point.

**Fig 3:**
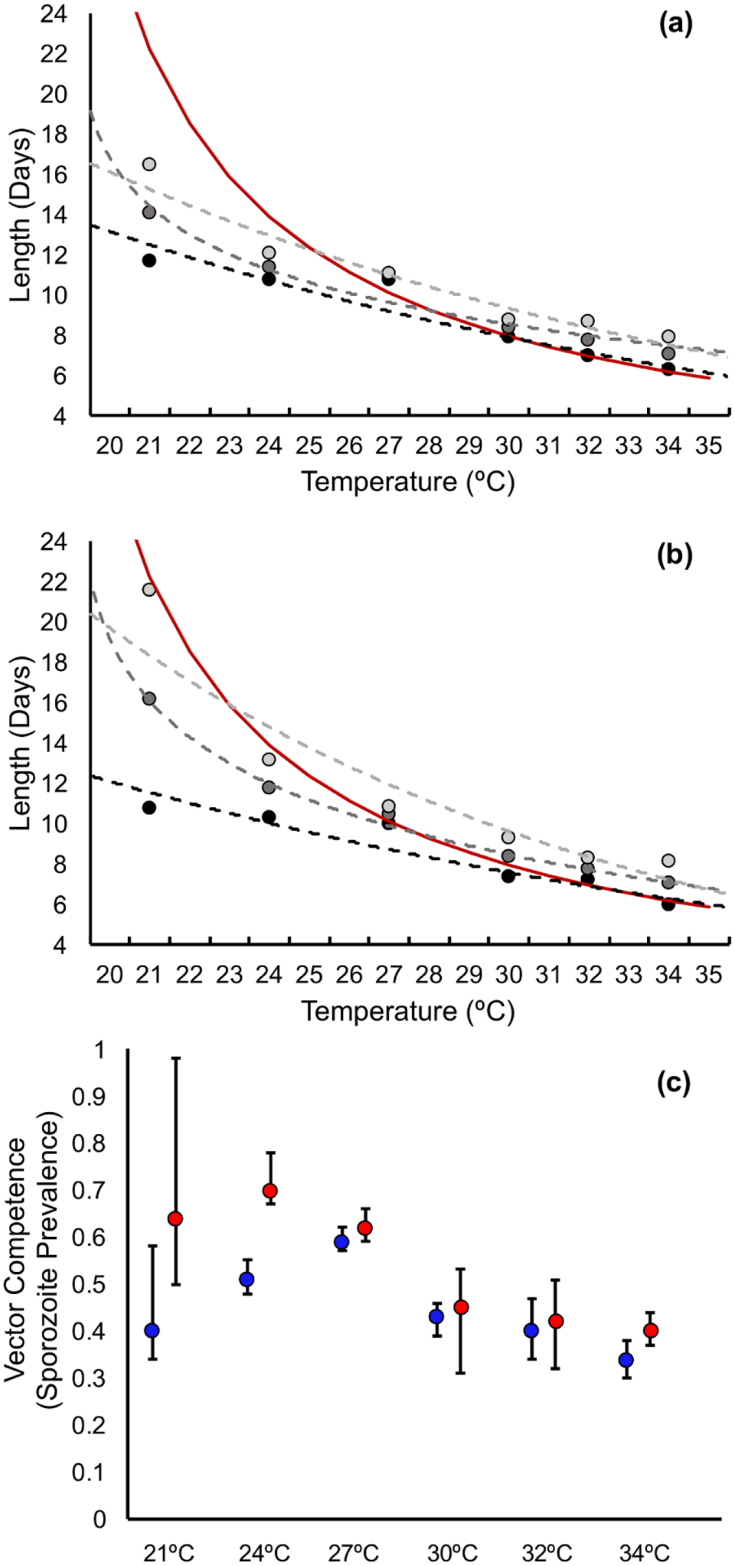
Predicted values for EIP_10_ (light grey), EIP_50_ (grey), and EIP_90_ (black) for each temperature in (A) experimental block 1 and (B) experimental block 2; dotted lines represent the predicted thermal performance curve for the respective EIPs, while the red line is the EIP of *P*. *falciparum* predicted from the widely-used degree-day model of Detinova 1962 [27]. (C) Predicted values for vector competence (g, the asymptote of the sporogony curve in Fig 2) across temperature for block 1 (blue) and block 2 (red). Error bars represent 95% confidence intervals. Numerical values can be accessed at http://dx.doi.org/10.5061/dryad.74389 [34]. EIP, extrinsic incubation period.

EIP is very poorly defined in the literature [4,47–54], so we provide here 3 estimates for each temperature to represent a range of possible interpretations of EIP (i.e., time in days to 10%, 50%, and 90% of maximum infectiousness). In Fig 3, we show how EIP_10_, EIP_50_, EIP_90_, and vector competence (maximum proportion infectious, or *g*, the asymptote of the logistic curve) values change with temperature in each experimental block (values for logistic model parameters are given in S10 Table). EIPs were shortest at 34°C and increased at cooler temperatures, irrespective of the specific measure of EIP (i.e., EIP_10_ increased from an average of 6.1 to 11.2 days, EIP_50_ increased from an average of 7.0 to 15.1 days, and EIP_90_ increased from an average of 8.0 to 19.0 days; see S11 Table for details). Additionally, the relative and absolute difference between EIP_10_ and EIP_90_ increased progressively under cooler conditions (Figs 2, 3A and 3B).

### Gonotrophic cycle

**Fig 4:**
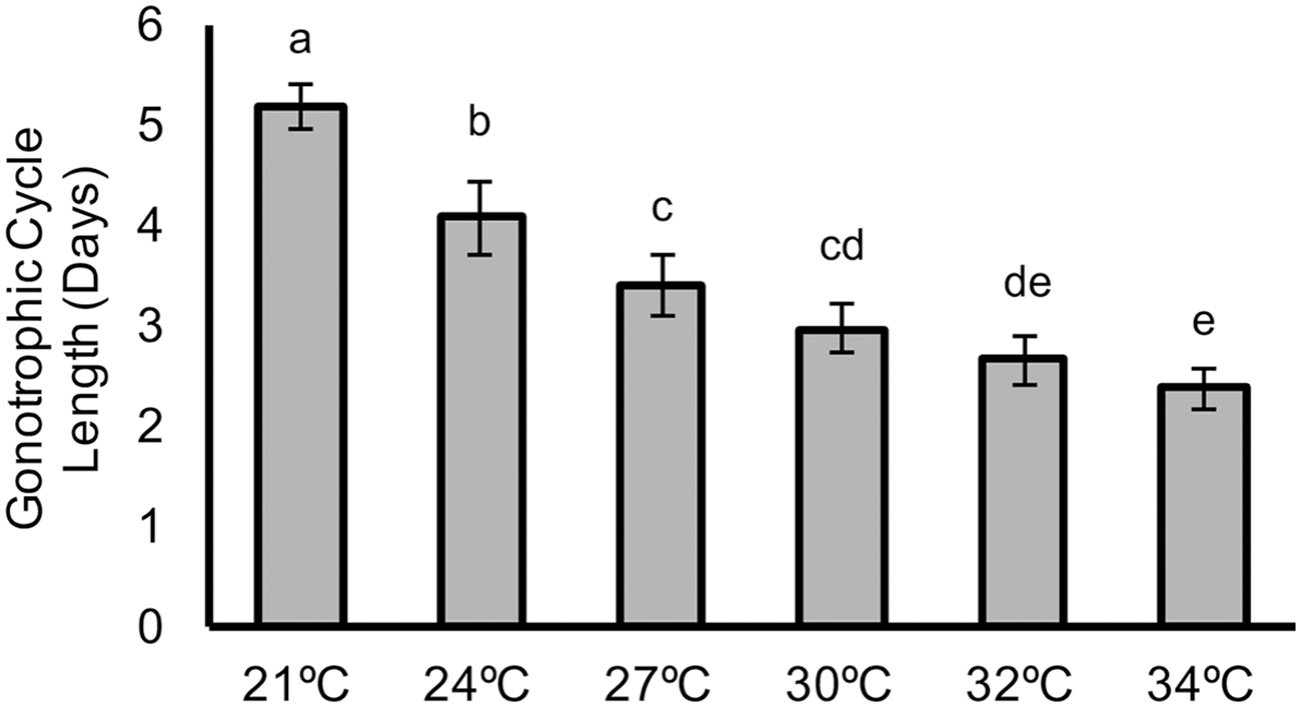
Mean length of the gonotrophic cycle (days) for each temperature. Error bars represent standard deviation; superscripts represent significant differences (*p* < 0.05) upon posthoc analysis. Numerical values can be accessed at http://dx.doi.org/10.5061/dryad.74389 [34].

We next conducted an experiment to determine the effect of temperature on the gonotrophic cycle length (i.e., the time from blood meal to oviposition), taking the mean of the first 2 gonotrophic cycles for each temperature. We found that gonotrophic cycle length declined with increasing temperature, although with differences between cycle lengths diminishing as temperature increased (Fig 4). The percentage of mosquitoes laying eggs was lower in the second gonotrophic cycle compared to the first, but there was no obvious effect of temperature on the likelihood of egg laying in either cycle (S12–S14 Tables).

### Thermal performance curves

Our empirical data enable us to generate thermal performance curves for biting rate, vector competence, mosquito mortality rate, and parasite development rate.

For estimates of daily biting rate, we followed convention by taking the reciprocal of the mean gonotrophic cycle length [51,52,54–57]. Some mosquitoes, such as *An*. *gambiae* and *An*. *funestus*, have been shown to take multiple blood meals per gonotrophic cycle [58]. However, there are no data from the field to suggest this behavior for *An*. *stephensi*.

For vector competence, we used values of the asymptote (*g*) of our logistic curves, while for parasite development rate we used the reciprocal of the median EIP (EIP_50_).

Generating a thermal performance curve for daily mosquito mortality rate is challenging, as we show mortality rate to increase with mosquito age. Accordingly, we follow the methodology described in [4] to fit negative exponential functions to the beginning and end points of the Gompertz distributions and use the exponent to approximate a fixed daily mortality rate for each block and temperature combination (see electronic supplementary material for further methodology and accompanying datasets and figures).

**Fig 5:**
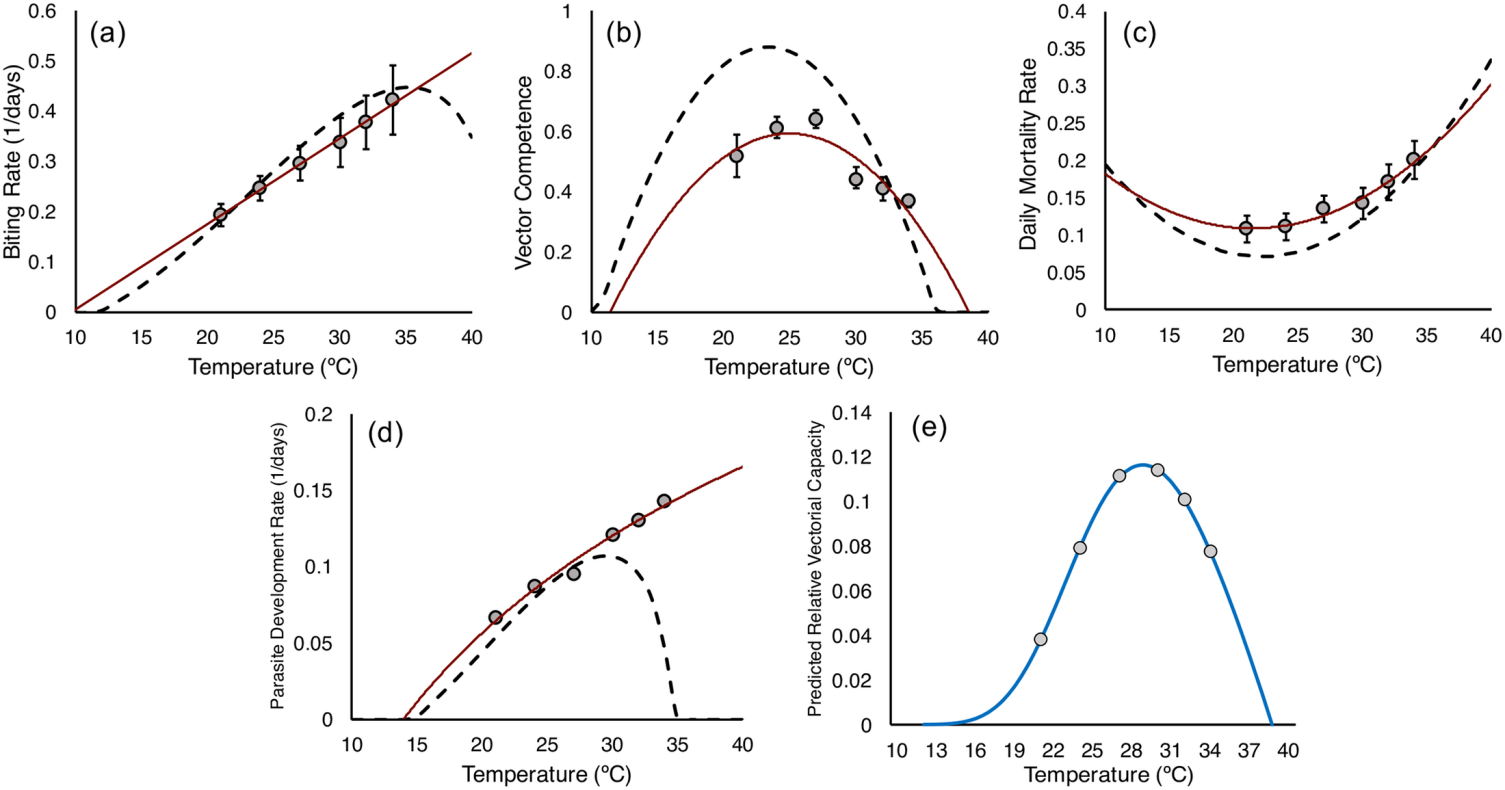
Thermal performance curves for (A) biting rate, (B) vector competence, (C) mosquito mortality rate, and (D) parasite development rate (based on the extrinsic incubation period time in days until 50% of maximum infectiousness [EIP_50_]), comparing the current study to the equivalent curves proposed by Mordecai et al. [4] by using mixed-species data. (E) Shows the predicted temperature-dependent model of rVC based on the thermal performance curves from this study, using data for the EIP_50_. Numerical values can be accessed at http://dx.doi.org/10.5061/dryad.74389 [34]. EIP, extrinsic incubation period; rVC, relative vectorial capacity.

We present the thermal performance curves for these traits in Fig 5, together with equivalent thermal performance curves from the study of Mordecai et al. [4]. Our thermal performance curves exhibit quantitative and qualitative differences to the established thermal performance curves derived from mixed-species data (for additional information comparing specific nonlinear models between this paper and those published previously, see S16 Table).

### Transmission potential

Having defined the effects of temperature on biting rate, mortality rate, EIP, and vector competence, it is possible to characterize the overall effects of temperature on transmission potential using a metric such as rVC. The rVC is the daily rate at which mosquitoes can transmit parasites to humans (relative to the vector-to-human population ratio, which here we do not define) [52–54, 56, 59], and is described by the following equation:

$$rVC=\;\frac{a^{2}be^{\left(-\mu n\right)}}{\mu }$$

where *a* is the daily biting rate, *b* is vector competence, *μ* is the daily mosquito mortality rate and *n* is the length of the EIP_50_ (see S2 Fig, S15 Table for calculations of rVC using EIP_10_ and EIP_90_ as alternatives).

**Fig 6:**
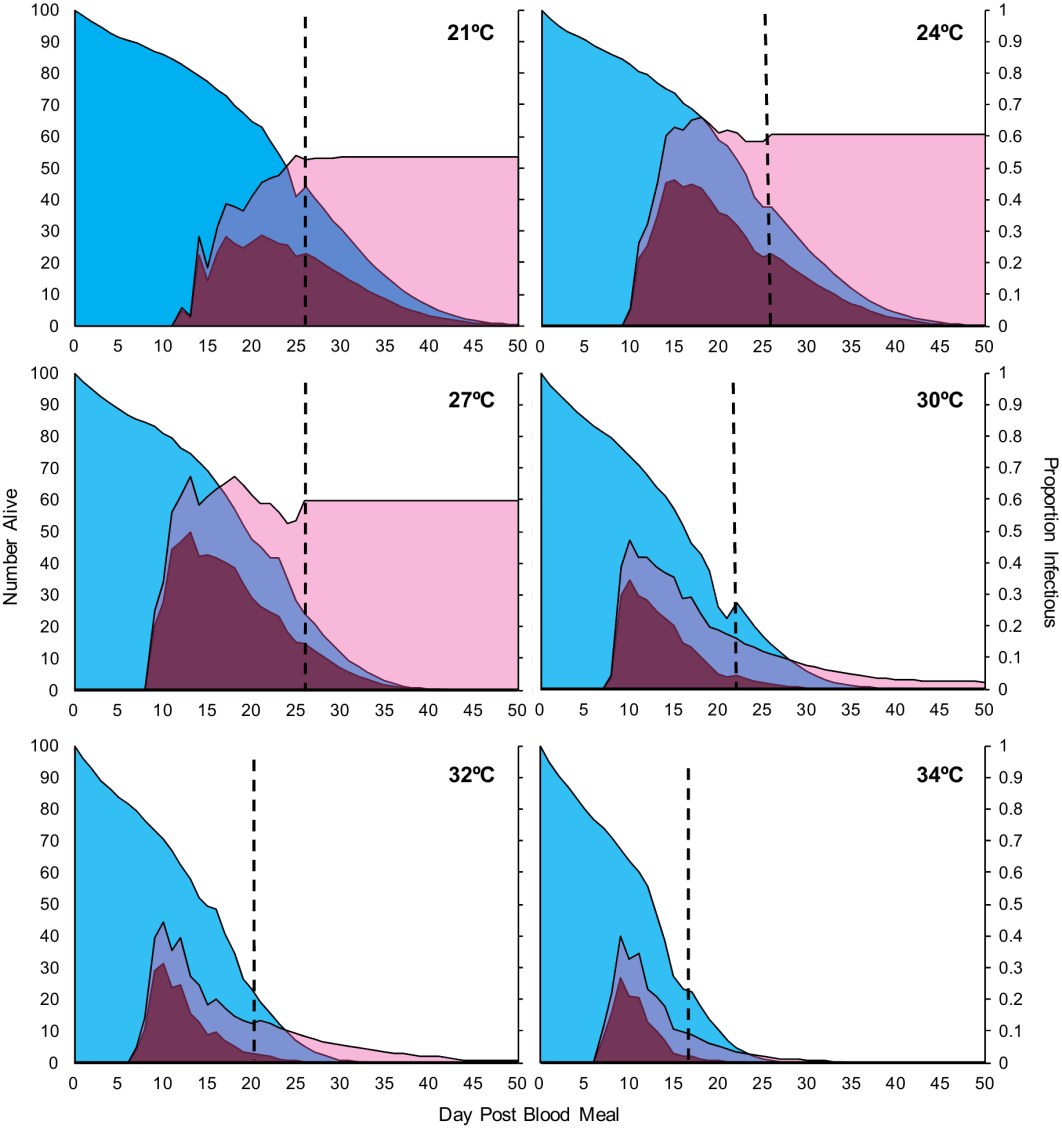
Curves for dynamic model of transmission potential for each temperature. Area curves for rates of survival (blue) and infection (pink) for each temperature; pPurple areas represent the product of the two 2 curves (i.e., the number of mosquitoes alive and infectious or “infectious mosquito days”). Dashed line represents the day at which collection of raw data ended; curves to the right of the dashed line represent values calculated from both survival and infection model estimates (mean for both experimental blocks). Numerical values can be accessed at http://dx.doi.org/10.5061/dryad.74389 [34].

In Fig 5E we show the temperature-dependent model of rVC based on our thermal performance curves. This model suggests a temperature optimum for transmission of 29°C, with an upper maximum threshold of 38°C and a minimum of 12°C. However, standard vectorial capacity models [1, 53–57, 59] and related models such as the basic reproductive rate, R_0_ [4, 51, 60], assume constant mortality rate per day, a discrete value for EIP at a given temperature, and no change in the proportion of infectious mosquitoes over time (so, no recovery from infection or altered survival rates due to infection). Our empirical data violate these assumptions, and so we also explore the effects of temperature on relative transmission potential using an alternative measure, adapted from the work of Killeen et al. [61] (see also [45,46] for similar methods). This model explicitly uses the full distributions for survival, sporogony, and competence by multiplying the number of mosquitoes alive on any given day (values from our survival curves) by the probability of being infectious (values from our curves for change in proportion of infectious mosquitoes over time). The product of these 2 proportions (area under the intersection of the 2 curves in Fig 6) represents the daily number of infectious mosquitoes, which we term “infectious mosquito days.” The number of infectious mosquito days is then multiplied by our empirical estimates of biting rate for each temperature to give the probable number of infectious bites transmitted by a cohort of mosquitoes over a given time period, assuming all blood meals are taken on humans; this is analogous to the relative force of infection.

Because our survival data were truncated, we extended our survival estimates out to day 50, which is the point at which mosquito survival dropped below 1% in the longest-lived temperature treatment. For each temperature, we used the mean of the daily values calculated from the empirical survival and data from both blocks, and used the model fits only for the tails of distributions where we have no raw data.

In Table 2, we provide values for the predicted number of infectious bites transmitted by a cohort of 100 mosquitoes over a period of 50 days for each temperature (see S3 Fig for schematic outlining the model approach).

**Table 2:** Predicted number of infectious bites for a cohort of 100 females over a period of 50 days.

Temperature	Infectious Mosquito Days	Biting Rate	Predicted Bites
21°C	493.4	**0.193** (0.171–0.215)	**95.2** (84.4–106.1)
24°C	695.9	**0.246** (0.222–0.270)	**171.2** (154.5–187.9)
27°C	630.4	**0.296** (0.261–0.331)	**186.6** (164.5–208.7)
30°C	257.3	**0.337** (0.288–0.386)	**86.7** (74.1–99.3)
32°C	196.8	**0.377** (0.324–0.430)	**74.2** (63.7–84.6)
34°C	133.0	**0.421** (0.352–0.49)	**56.0** (46.8–65.17)

Mean (and standard deviation) number of infectious bites predicted for a cohort of 100 females over a period of 50 days. Values calculated by using the relative force of infection model that takes into account the dynamic distribution of both mortality and infection and daily biting rate.

**Fig 7:**
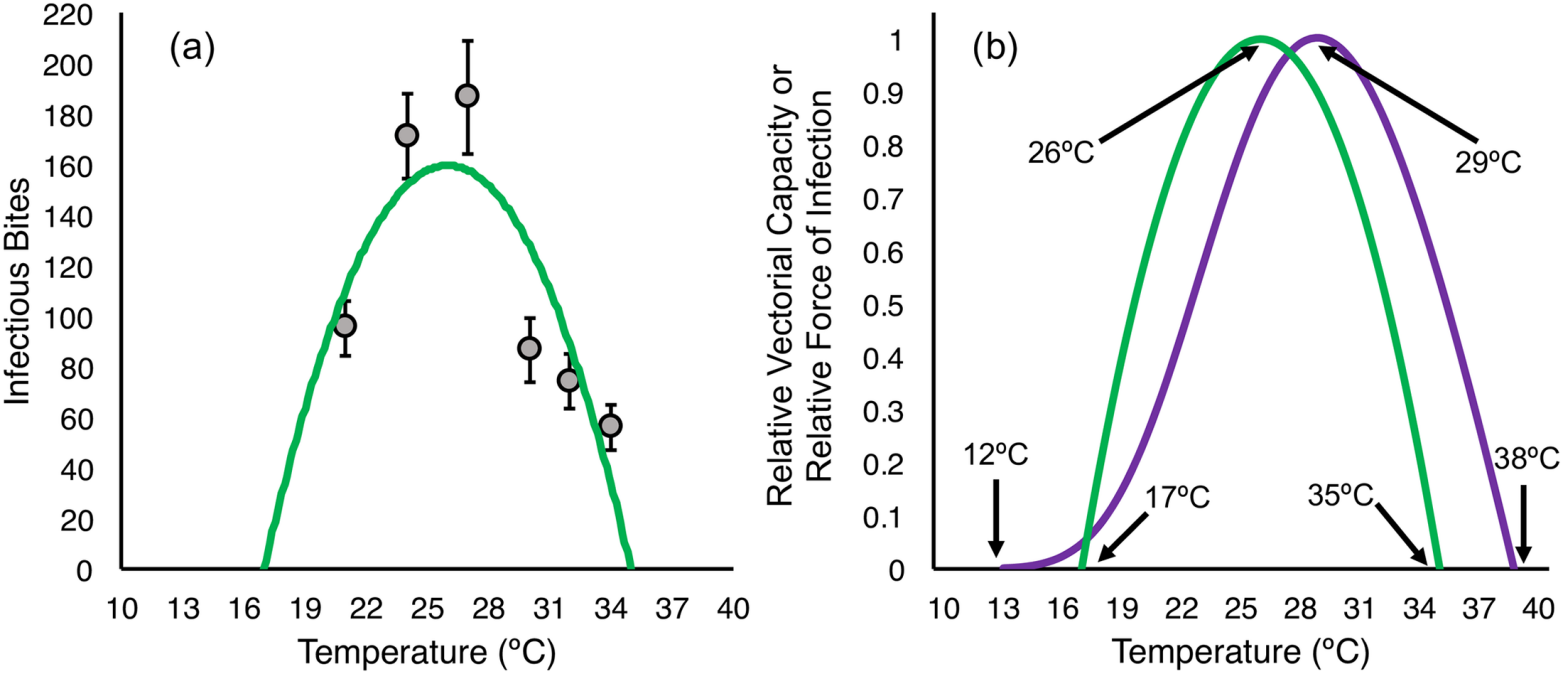
(A) Best fit thermal performance curve for relative force of infection (here the number of infectious bites predicted for a cohort of 100 female mosquitoes); grey points represent the calculated number of bites for the mean of both experimental blocks and error bars represent standard deviation. (B) Comparison of scaled thermal performance curves for rVC and relative force of infection. Numerical values can be accessed at http://dx.doi.org/10.5061/dryad.74389 [34]. rVC, relative vectorial capacity.

We then fit a nonlinear curve (in this case the best fit model was a simple quadratic function) to our data points for mean transmission potential at each temperature (Fig 7A). The resultant thermal performance curve of relative force of infection suggests a temperature optimum of 26°C, with lower and upper thresholds of 17°C and 35°C, respectively. In Fig 7B, we compare the models of rVC and relative force of infection.

## Discussion

This study represents one of the most detailed empirical investigations of the effects of temperature on *P*. *falciparum* and a key mosquito vector conducted to date. The results yield several important insights that challenge classical assumptions. We also provide an experimental blueprint for future species-specific explorations of temperature-mediated changes in malaria transmission, and such data will be essential for future predictive and theoretical modeling studies.

Most conventional malaria transmission models assume constant rate of adult mortality per day [1, 4, 50, 53–55, 59]. In contrast, we show that mosquito mortality rate is most accurately described using an age-dependent distribution, in this case, a Gompertz function. Other studies also suggest age-dependent survival (e.g., [40, 62–65]). Here, we show that the age-dependence holds across temperatures, with initial senescence rate (α) increasing at higher temperatures. We acknowledge that ours is a lab-based study and that the empirical survival data were necessarily truncated because of destructive sampling of mosquitoes for dissection (i.e., Kaplan-Meier estimates are interval-censored). Future studies would benefit from monitoring survival in a parallel group of mosquitoes that receive the same infectious blood meal but with no samples removed for dissection.

A recent analysis of survival data for *Aedes* mosquitoes indicated that age-dependent mortality is more likely to be found in the laboratory as external mortality factors such as predation, encountering insecticides, and nutritional stress tend to be more prevalent in the field, confounding the effects of senescence [40]. However, a previous field study on geographically-distinct populations of *A*. *aegypti* in Southeast Asia and Latin America suggests that age-dependent mortality is observable in the field, and that older mosquitoes die at quicker rates than younger mosquitoes in the same cohort [35]. Whether our data are an artifact of prolonged survival under laboratory conditions is unclear, as very few data exist on the nature of the mortality distributions of adult *Anopheles* spp. under field conditions.

Existing models of *P*. *falciparum* sporogony, either the classic degree-day models [27, 28] or more contemporary thermal performance curves [4], provide a single estimate of EIP for a mosquito population at a given temperature. We show that sporogony is not perfectly synchronized between individual mosquitoes, but instead follows a distribution across the mosquito population over time, with temperature affecting the extent of the distribution (i.e., the number of days over which sporogony occurs), as well as the median value (see also [1, 46]). Most empirical studies are vague in reporting whether they are defining the EIP as the first mosquitoes to become infectious (approximating our EIP_10_), the median value (EIP_50_), or the time to maximum prevalence (approximating our EIP_90_). Our results clearly show potential for substantial variation between these measures, particularly at cooler temperatures. In turn, these discrete, single-value measures of EIP can yield markedly different estimates of transmission potential (such as rVC) for the same mosquito population (S2 Fig, S15 Table).

A number of recent studies describe parasite development rate (the reciprocal of the EIP) as a unimodal function, suggesting a decline in growth rate as temperatures increase above the optimum [4, 25, 66]. The unimodal functions result from inclusion of data (often single data points) at high temperatures in which parasites fail to complete development. Yet, there is a substantial mechanistic difference as to whether high temperatures limit transmission because parasite survival/vector competence declines or because parasite growth slows and EIP becomes progressively long (and is assumed to be infinite at the point where no parasites survive). We find no evidence of an increase in EIP at high temperatures. More data are needed to resolve this fundamental issue.

Furthermore, at high temperatures we see a decline in the prevalence of potentially infectious mosquitoes over time, suggesting either that mosquitoes are recovering from infection (i.e., sporozoites are dying or otherwise being cleared from the salivary glands and surrounding hemolymph), or that infectious mosquitoes exhibit differential mortality and die at a quicker rate compared with noninfectious mosquitoes. Our current experimental design does not enable us to determine the mechanism explicitly, but when we compare overall mosquito survival with the rate of decline in the proportion of infectious mosquitoes over time, we see no significant difference in instantaneous hazard rates (S16 Table, S4 and S5 Figs, see supplementary methods for analysis). This outcome is more consistent with enhanced death of infectious mosquitoes rather than parasite clearance. It has been suggested that *P*. *falciparum* has no lethal effect on naturally occurring mosquito hosts [67], yet most studies examine malaria infections in the range of 24°C to 28°C. Our data suggest that *P*. *falciparum* might impact mosquito survival at higher temperatures (see also [7]). We are not aware of any malaria transmission models that consider possible costs of parasite infection under increased environmental stress (temperature or otherwise).

Finally, we show that the predicted effects of temperature on overall transmission potential differ between a standard vectorial capacity model and an alternative model of force of infection, with further differences within the vectorial capacity model that are dependent on which estimate of EIP is used (See Fig 2, S15 Table). One reason for this difference is that the empirical life history parameters contradict several of the model assumptions implicit in the vectorial capacity approach. These differences between models could have important biological significance. For example, the differences in the upper and lower thermal limits for transmission would generate different patterns of range expansion and contraction in response to climate change. Within the transmission range, a shift in temperature from 24°C to 28°C (e.g., via seasonal change or longer-term climate warming) would be predicted to increase rVC by 34% but decrease relative force of infection by 1%. At the thermal extremes, even small shifts in temperature have quantitatively different outcomes; warming from 32°C to 34°C, for example, reduces rVC by 30% but reduces the force of infection model by 62%.

Our model of rVC derives from the standard formulation developed by Garret-Jones [53], which is itself a simplified version of the original dynamical model as developed by Macdonald [47]. Alternative formulations of vectorial capacity, such as presented in Brady et al. [68], could generate different thermal response curves for transmission as they combine individual life history traits in different ways. Extension of either the rVC model or the model of relative force of infection to a more holistic metric, such as the basic reproductive rate, requires additional information on actual mosquito density (also likely temperature dependent through effects of temperature on larval development rate and survival [66]), as well as susceptibility and rate of recovery of the human host. Regardless of model framework, properly characterizing the thermal performance curves for individual traits remains important, especially for key traits such as the proportion of mosquitoes surviving the EIP, or the frequency of blood feeding, as these are integral to transmission.

In general, we demonstrate that a detailed, system-specific examination of temperature sensitivity yields quantitatively and qualitatively different estimates of temperature-dependent life history traits, compared to the currently accepted relationships that integrate data from diverse studies and a mixture of mosquito and parasite species. For some traits, the differences appear small (e.g., our data on biting rate match previous data quite closely, at least over the temperature range of the current study). For other traits, the differences are substantial; our observed mortality rates are much higher, our parasite development rates are greater than those predicted by standard models at cool temperatures, and unlike contemporary unimodal thermal performance curves [4], we see no evidence for an increase in EIP at high temperatures.

We acknowledge that malaria transmission is not determined by temperature alone [55, 69–71]. Furthermore, we used long-standing lab colonies of a single mosquito–parasite combination; it is likely that parasite development, vector competence, biting rate, and longevity vary between species and between local populations [7, 57, 62, 72, 73], including the potential for local thermal adaptation [72, 74]. Yet, there is little reason to think that our system is more idiosyncratic than any other local malaria vector-parasite pairing in nature. We also focus on describing the effects of constant temperatures, as this is consistent with nearly all other studies to date. However, our own research has shown that daily variation in temperature can influence mosquito and parasite life history traits above and beyond the effects of mean temperature alone [2, 12, 66]. Future studies would benefit from the inclusion of daily temperature variation, particularly at high and low temperature extremes, as variation is likely to play an important role in defining the upper and lower limits of transmission. Inclusion of variation in biotic factors, such as differences in larval habitat quality, would also be valuable as these can further shape transmission potential [46, 75–77]. Such ecological complexities only strengthen the need for more detailed, system-specific studies of the type presented here in order to fully understand the influence of temperature on transmission and generate more informed predictions of the potential impact of climate change.

## Materials and methods

### Temperature selection and maintenance

Temperatures were selected to capture key transmission range for *P*. *falciparum* [1, 4, 27, 28, 30]. All mosquitoes were housed in secure cardboard cups inside secondary mesh containment cages. Cages were kept in environmentally controlled reach-in incubators at 21°C, 24°C, 27°C, 30°C, 32°C, and 34°C, each ± 0.5°C and 80% ± 5% relative humidity. To ensure incubators were functioning correctly, we placed battery-powered portable USB dataloggers in each incubator. Daily data were extracted at approximately 9:00 am to ensure temperature and humidity were stable throughout the duration of the experiment.

### Plasmodium falciparum culture and infection

In vitro cultures of *P*. *falciparum* strain NF54 (wild type, Center for Infectious Disease Research, Seattle, Washington) were maintained in RPMI 1640 medium (25 mM HEPES, 2 mM L-glutamine), supplemented with 50 μM hypoxanthine and 10% human A+ serum (Valley Biomedical, Winchester, Virginia). Culture was maintained in an atmosphere of 5% CO_2_, 5% O_2_, and 90% N_2_. Parasite cells were then subcultured into O+ human erythrocytes (Valley Biomedical). Gametocyte initiation occurred at 5% haematocrit and 0.8% to 1.0% mixed-stage parasitemia. The culture was maintained for 17 days and parasite cells were provided fresh media daily.

On the day of the blood meal, gametocyte cultures (approximately 8% gametocytemia for each experimental block) were briefly centrifuged, and the supernatant was removed and discarded. Pelleted erythrocytes were diluted to 40% haematocrit using fresh A+ human serum and O+ human erythrocytes. The mixture was pipetted into glass bell jars fixed with a Parafilm membrane and connected by plastic tubing with continuously flowing water heated to 37°C. Each bell jar was filled with 2 mL of blood culture, which feeds approximately 200 females. Mosquitoes were given 20 minutes to fully engorge, after which the bell jars were removed, as the parasites in culture are no longer viable after 20 minutes. In each cup, >95% of females were observed to have engorged fully. Immediately following the blood meal, mosquitoes were transferred to their respective temperature treatments and maintained by providing cotton balls soaked with 10% glucose and 0.05% para-aminobenzoic acid in water, which were replaced daily.

### Parasite development and mosquito survival

*An*. *stephensi* Liston adult females were from our laboratory colony (originally derived from a long-standing colony at the Walter Reed Army Institute of Research, Silver Spring, Maryland) maintained at standard insectary conditions (27°C ± 0.5°C, 80% ± 5% relative humidity and a 12:12 photoperiod). Three- to 5-day-old females were aspirated into cardboard cups (475 mL), with approximately 150 per cup. Four cups were allocated to each of the 6 incubators set at the different experimental temperatures (21°C, 24°C, 27°C, 30°C, 32°C, and 34°C), totalling approximately 600 females per temperature. Each cup was provided a human blood meal infected with in vitro cultured *P*. *falciparum* strain NF54 (wild type, Center for Infectious Disease Research).

Salivary gland sampling began on day 10 post-blood meal for 21°C and 24°C, day 8 for 27°C, day 6 for 30°C, and day 5 for 32°C and 34°C, based on results from a pilot experiment (S1 Fig). For each sample, 8 to 10 mosquitoes were aspirated from each replicate cup into absolute ethanol and salivary glands were dissected. Glands were ruptured beneath a glass cover slip and examined under a light microscope at 40x for presence of sporozoites.

Dead mosquitoes were counted daily in each cup. For survival analysis, mosquitoes removed for dissections each day and those that remained alive at the end of the experiment (day 25) were considered censored cases. We compared a range of plausible mortality curves including exponential, log-logistic, log-normal, Weibull, and Gompertz distributions for each block x temperature combination individually by using the R package flexsurv and selected the best-fit model using AIC (S7 and S8 Tables).

### Gonotrophic cycle length and biting rate

To estimate effects of temperature on the length of the gonotrophic cycle, 3- to 5-day-old females were fed to repletion on a membrane feeder using pork intestine sausage casing filled with human blood heated to 37°C. Fully engorged females (*n* = 85 per temperature treatment) were then transferred to individual 50 mL plastic tubes covered with mesh and filled with 5.0 mL of distilled water. Each tube was provided a cotton ball moistened with 10% glucose solution that was replenished daily. Daily, tubes were checked for presence of eggs between 9:00 am and 10:00 am. Females in tubes that had laid eggs were then transferred to a clean tube and sugar was withheld for 6 hours, after which these females were offered a second human blood meal on the membrane feeding system (in groups of 5 tubes per feeder, all feeds occurred at 27°C for optimum response).

This allowed for a quantification of the length of time to laying the first and second clutches; the mean of these values was used as gonotrophic cycle length. For females not laying a second clutch, the number of days to laying the first clutch was considered as the mean in the final calculation of mean cycle length. To calculate biting rate, we used the reciprocal of the mean gonotrophic cycle for each temperature. Differences in biting rate were assessed using a Kruskal-Wallis test followed by Dunn’s post-hoc rank sum comparison using the R package pgirmess.

To assess if temperature affected the likelihood of egg laying in general, mating success was also assessed by dissection of spermathecae from females in that had not laid eggs by day 21 post-blood meal. Spermathecae were ruptured under a glass cover slip and observed under a light microscope at 40x magnification. Presence of sperm, whether alive or dead, was considered a successful mating. Data on each individual clutch and mating success can be accessed in the supplementary materials (S12–S14 Tables).

Raw data for survival and infection, R script for statistical analysis, and numerical values for producing each figure can be accessed in the Dryad data repository: http://dx.doi.org/10.5061/dryad.74389 [34].

## Supporting information

S1 Fig Experimental design schematic. (TIFF)

S2 Fig Dynamic transmission potential model schematic. (TIF)

S3 Fig Dynamics of survival and infection at 30°C. Dynamics of survival (purple line) and the proportion of mosquitoes alive and infectious (orange line) for each replicate cup and block in the 30°C treatment used to mathematically analyze the possibility of differential mortality.
(TIF)

S4 Fig Dynamics of survival and infection at 32°C. Dynamics of survival (purple line) and the proportion of mosquitoes alive and infectious (orange line) for each replicate cup and block in the 32°C treatment used to mathematically analyze the possibility of differential mortality. Replicate cup 4 in experimental block 1 was discarded due to sugar pad being replaced with a water pad after blood feeding, so unusually high mortality due to starvation occurred during the first 24 hours post-blood meal.
(TIF)

S5 Fig Dynamics of survival and infection at 34°C. Dynamics of survival (purple line) and the proportion of mosquitoes alive and infectious (orange line) for each replicate cup and block in the 32°C treatment used to mathematically analyze the possibility of differential mortality. Replicate cup 4 in experimental block 1 was discarded due to sugar pad being replaced with a water pad after blood feeding, so unusually high mortality due to starvation occurred during the first 24 hours post-blood meal.
(TIF)

S1 Table Data from each temperature group from pilot experiment. Data for proportion of infectious mosquitoes on each day of salivary gland dissections during pilot experiment.
(XLSX)

S2 Table Pairwise log-rank tests between blocks across temperature. Results of post-hoc pairwise log-rank statistics across all temperatures, comparing temperatures between block.
(XLSX)

S3 Table Pairwise log-rank tests between temperature across both blocks. Results of post-hoc pairwise log-rank statistics across both blocks, comparing all temperatures to each other across blocks.
(XLSX)

S4 Table Pairwise log-rank tests between temperatures within block 1. Results of post-hoc pairwise log-rank statistics across temperature in experimental block 1, comparing all temperatures to each other. Bold italics represent groups not statistically different from each other.
(XLSX)

S5 Table Pairwise log-rank tests between temperatures within block 2. Results of post-hoc pairwise log-rank statistics across temperature in experimental block 2, comparing all temperatures to each other.
(XLSX)

S6 Table Pairwise log-rank tests between temperature and block. Results of post-hoc pairwise log-rank statistics across temperature and block, comparing all temperatures to each other between each block. Bold italics represent groups not significantly different from each other.
(XLSX)

S7 Table Comparison of survival model distributions across block for 21°C, 24°C, and 27°C. Comparison of survival models built using the R package *flexsurv* using 3 survival distributions (Gompertz, Weibull, and exponential) commonly used in studies of mosquito mortality across block and temperature for 21°C through 27°C. Bolded models represent the best fit model for each block x temperature combination.
(XLSX)

S8 Table Comparison of survival model distributions across block for 30°C, 32°C, and 34°C. Comparison of survival models built using the R package *flexsurv*using 3 survival distributions (Gompertz, Weibull, and exponential) commonly used in studies of mosquito mortality across block and temperature for 30°C through 34°C. Bolded models represent the best fit model for each block x temperature combination.
(XLSX)

S9 Table Nonlinear models for post-truncated data for 30°C, 32°C, and 34°C. Nonlinear exponential models used for each block and temperature combination for instances where a decrease in proportion of infectious mosquitoes was observed. Models represent the description of the curve only for data points past the point of truncation (day of peak proportion of infectious mosquitoes) used for binary logistic models.
(XLSX)

S10 Table Parameters for binary logistic regression sporogony model. Parameter values (*g*, *k*, *t*_*m*_) for best fit model listed for and each block separately. Bolded numbers are predicted values, with 95% confidence intervals in parentheses.
(XLSX)

S11 Table Predicted EIP_10_, EIP_50_, and EIP_90_ values. Predicted values and 95% confidence intervals (in parentheses) for EIP_10_, EIP_50_, and EIP_90_ from the binary logistic regression model for each block and temperature combination as parameterized in S10 Table. EIP, extrinsic incubation period.
(XLSX)

S12 Table Gonotrophic cycle for first clutch of eggs. Data for number of females laying the first clutch of eggs and the mean gonotrophic cycle length for each temperature for the first clutch.
(XLSX)

S13 Table Gonotrophic cycle data from second clutch. Data for number of females still alive after laying the first clutch of eggs, the number that laid a second clutch of eggs, and the mean gonotrophic cycle length for each temperature for the second clutch of eggs.
(XLSX)

S14 Table Mating success data across temperatures. Mating success across each temperature in gonotrophic cycle/biting rate experiment. A successful mating was considered either successful oviposition or the presence of sperm in dissected spermathecae, in the case a female did not lay eggs over the course of the experiment.
(XLSX)

S15 Table Calculations for rVC using EIP_10_ and EIP_90_. Values for relative vectorial capacity using minimum estimate for EIP (defined here as EIP_10_, or time to 10% of maximum proportion infectious) and the maximum estimate for EIP (EIP_90_, or time to 90% of maximum proportion infectious). EIP, extrinsic incubation period.
(XLSX)

S16 Table Comparison of nonlinear models for performance of traits across temperature. Comparison of nonlinear models for performance of traits across temperature for the data presented in this paper and the data used in Mordecai *et al*. 2013. The R^2^ refers to our empirical data and the corresponding best fit models.
(XLSX)

S17 Table Differential mortality calculations. Mathematical analysis for the possibility of differential mortality or recovery. Each temperature x block x cup combination was analyzed using a Gompertz distribution with ‘surviving’ and ‘surviving and infectious’ as categorial covariates. Each combination was analyzed beginning at the day of peak prevalence and ending at the day of lowest prevalence. In the instance that two days in a row had the same peak prevalence, analysis began at the second day with the same value. Overlapping hazard rates (signified by “yes”) indicate that it is most likely that the decrease in the proportion of infectious mosquitoes in higher temperatures over time scales with mortality rates.
(XLSX)

S1 Text Calculation of negative exponential mortality rates for relative vectorial capacity from reduced Gompertz curves. (DOCX)

## Abbreviations

AIC: Aikake information criterion
EIP: extrinsic incubation period
rVC: relative vectorial capacity
