# Supplementary figures and images for: Quantifying the effects of temperature on mosquito and parasite traits that determine the transmission potential of human malaria
Source: PLoS Biol. 2017 Oct 16;15(10):e2003489. doi: 10.1371/journal.pbio.2003489 (PMC5658182; doi:10.1371/journal.pbio.2003489)

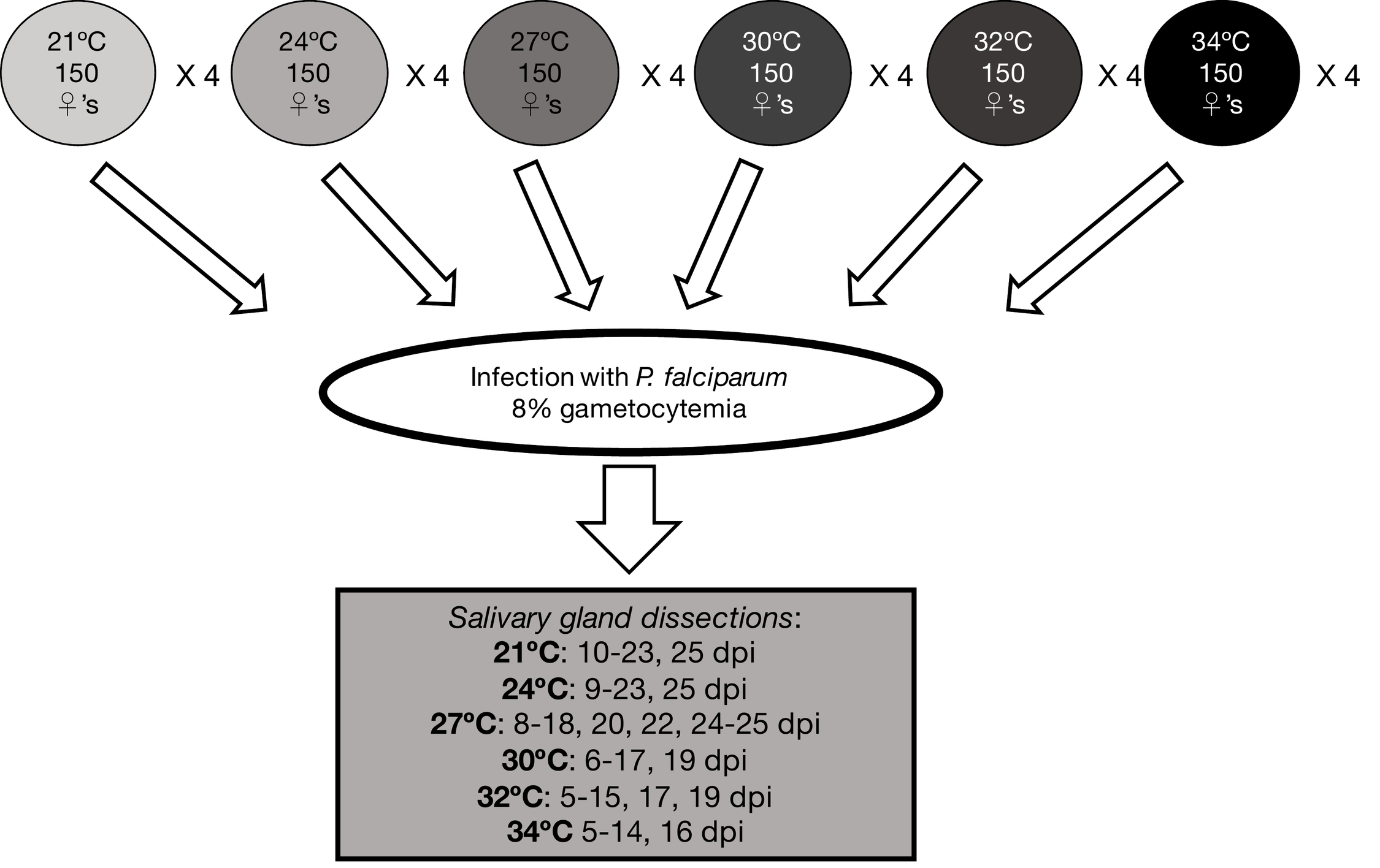

Supplement: S1 Fig — (TIFF) [file pbio.2003489.s001.tiff]

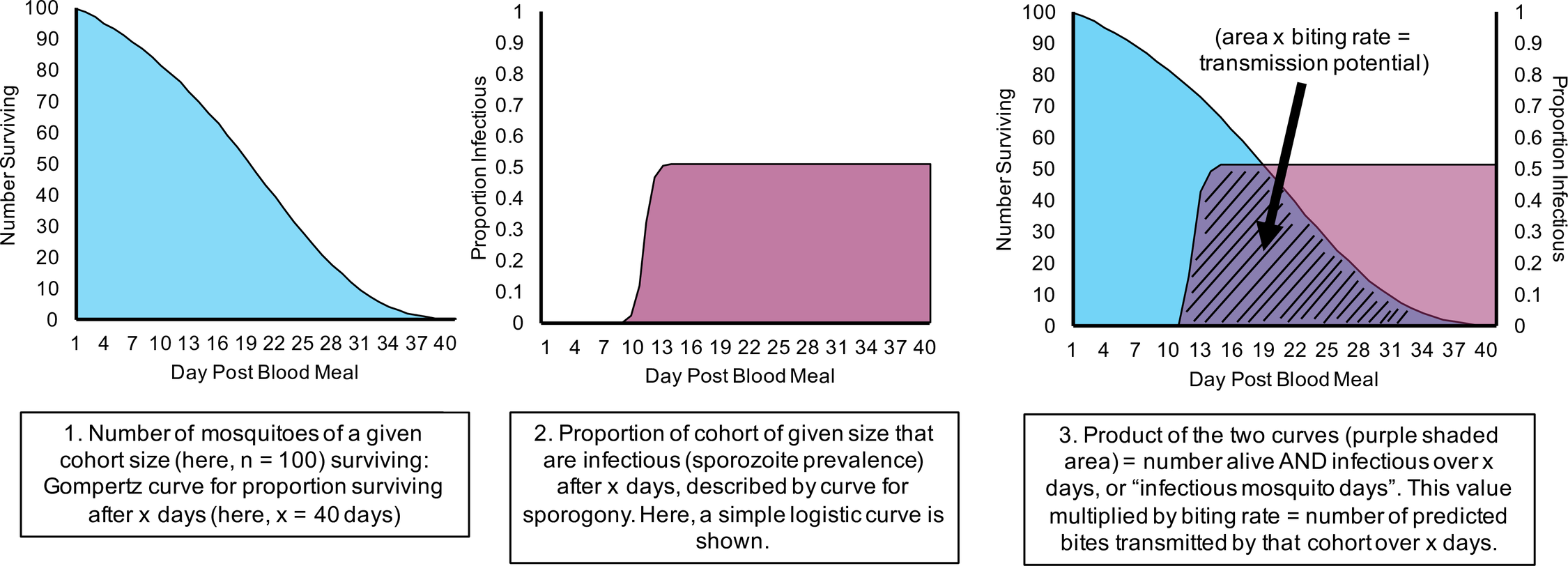

Supplement: S2 Fig — (TIF) [file pbio.2003489.s002.tif]

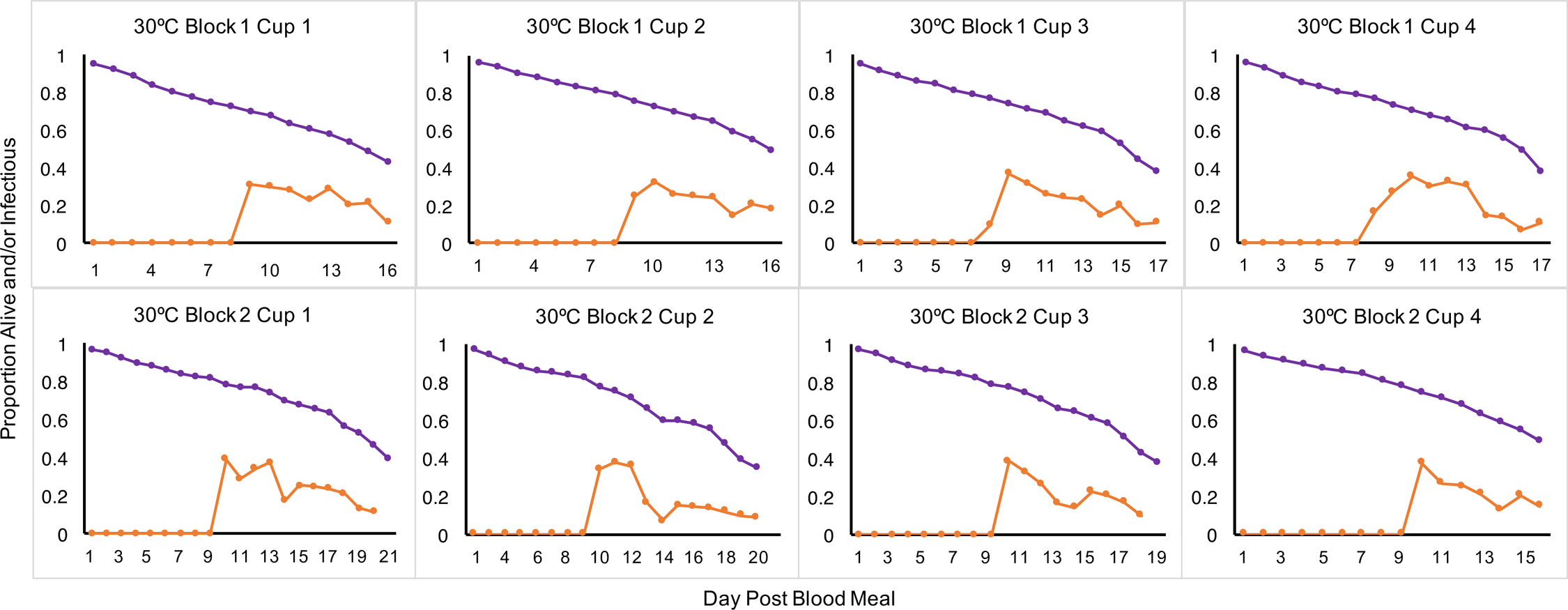

Supplement: S3 Fig — Dynamics of survival (purple line) and the proportion of mosquitoes alive and infectious (orange line) for each replicate cup and block in the 30°C treatment used to mathematically analyze the possibility of differential mortality. (TIF) [file pbio.2003489.s003.tif]

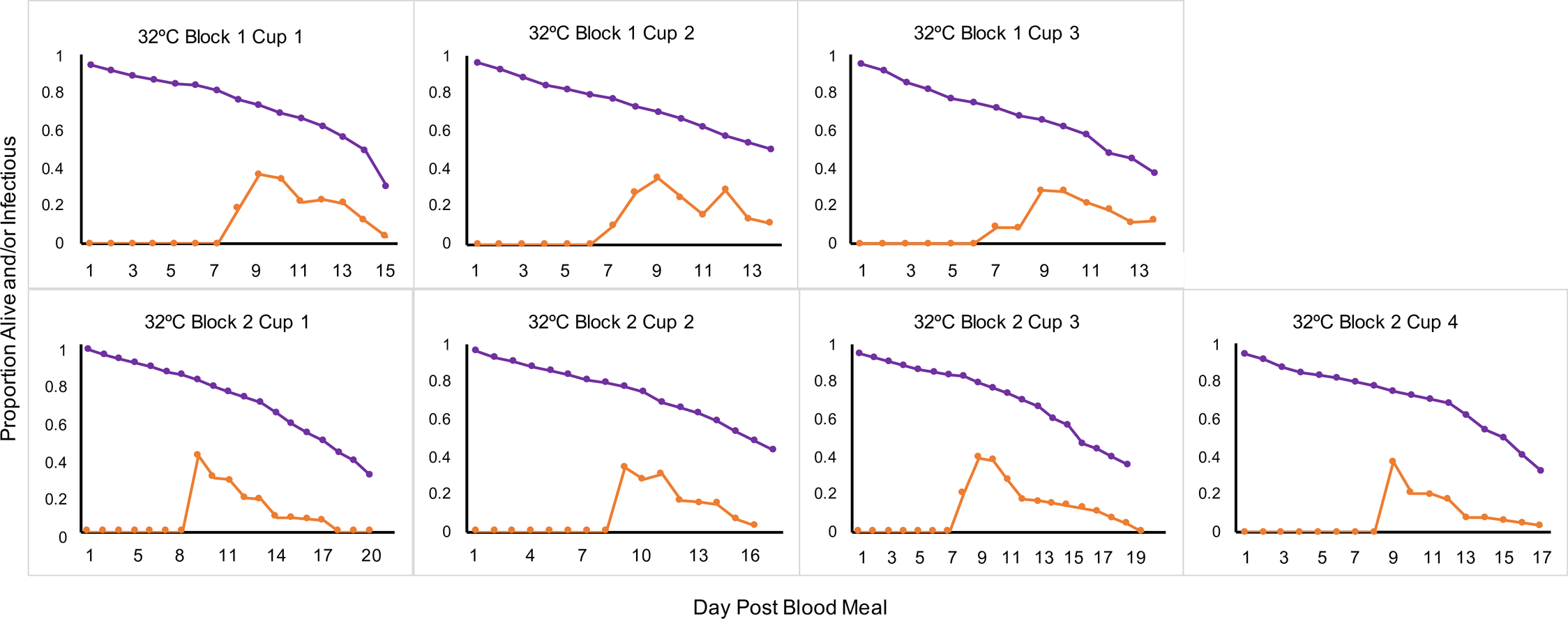

Supplement: S4 Fig — Dynamics of survival (purple line) and the proportion of mosquitoes alive and infectious (orange line) for each replicate cup and block in the 32°C treatment used to mathematically analyze the possibility of differential mortality. Replicate cup 4 in experimental block 1 was discarded due to sugar pad being replaced with a water pad after blood feeding, so unusually high mortality due to starvation occurred during the first 24 hours post-blood meal. (TIF) [file pbio.2003489.s004.tif]

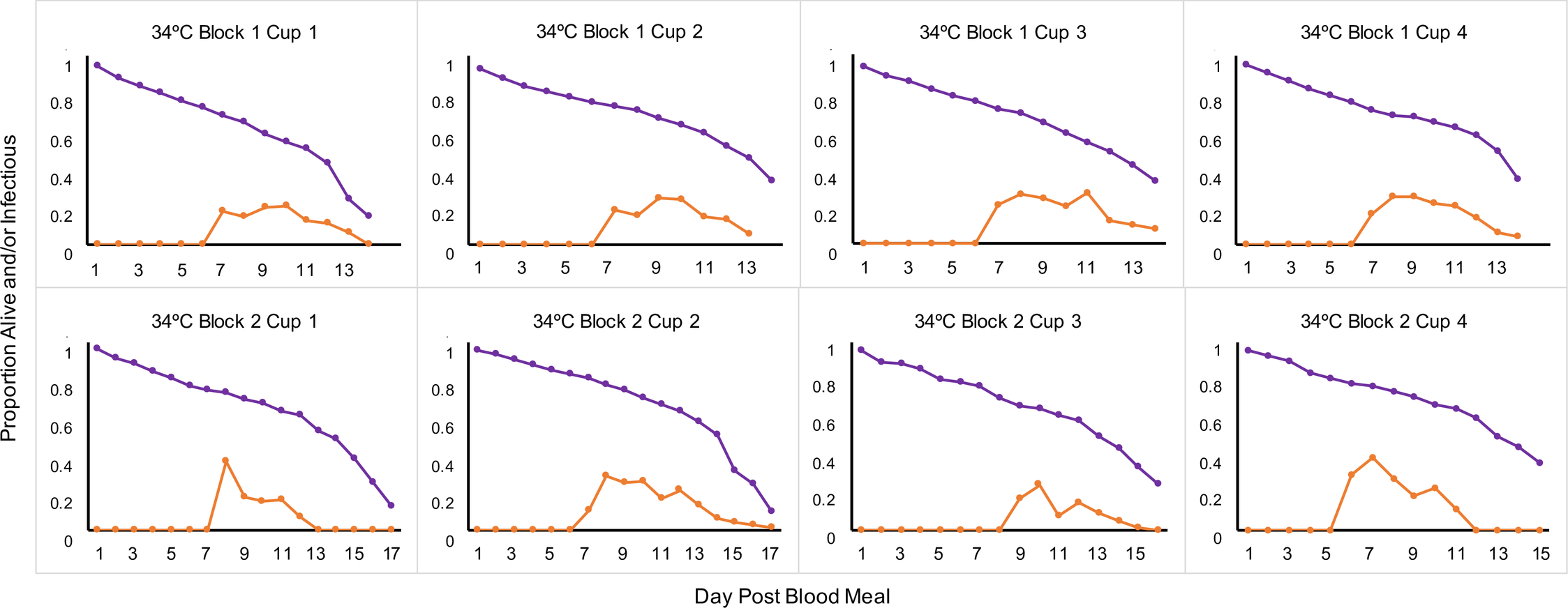

Supplement: S5 Fig — Dynamics of survival (purple line) and the proportion of mosquitoes alive and infectious (orange line) for each replicate cup and block in the 32°C treatment used to mathematically analyze the possibility of differential mortality. Replicate cup 4 in experimental block 1 was discarded due to sugar pad being replaced with a water pad after blood feeding, so unusually high mortality due to starvation occurred during the first 24 hours post-blood meal. (TIF) [file pbio.2003489.s005.tif]
