# Supplementary material for: Quantifying the effects of temperature on mosquito and parasite traits that determine the transmission potential of human malaria
Source: PLoS Biol. 2017 Oct 16;15(10):e2003489. doi: 10.1371/journal.pbio.2003489 (PMC5658182; doi:10.1371/journal.pbio.2003489)
Supplement: S1 Text — (DOCX) [file pbio.2003489.s023.docx]

**Methods for calculating exponential mortality rate from Gompertz data**

We calculated *μ* by estimating constant mortality rates by taking a subset of six data points from each Gompertz distribution for each block and temperature combination, to which we fit an exponential function, using the exponent of the constant for the function as our values for µ. We followed the same methodology as described in [4] to assign our data subset for each temperature and block combination: first, we used day 0 (survival = 1.0, or 100%), the day before survival probability was greater than 0.01 (or the nearest value), the day at which survival reached 0.01 (or the nearest value), and the three days following the 0.01 threshold. Below are the resulting tables and figures and equations from each temperature x block combination.

***21ºC Block 1***

***
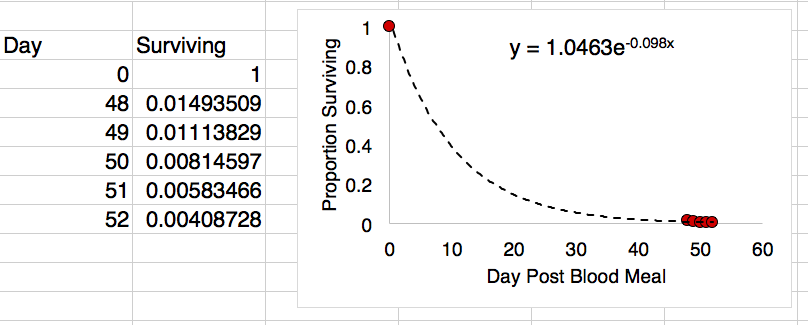
***

***21ºC Block 2***

***
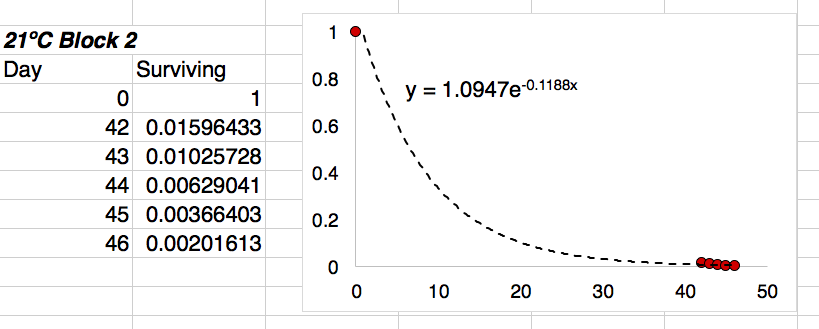
***

***24ºC Block 1***

***
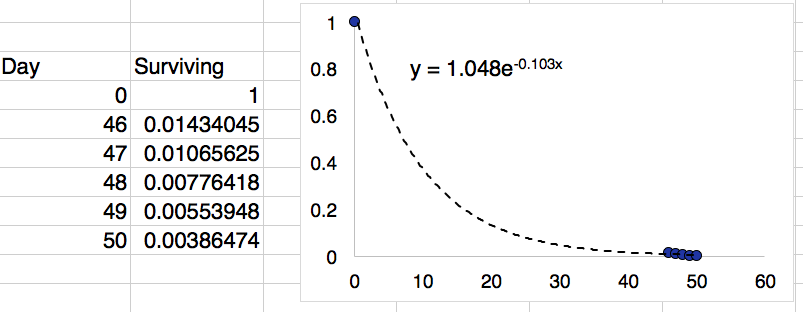
***

***24ºC Block 2***

***
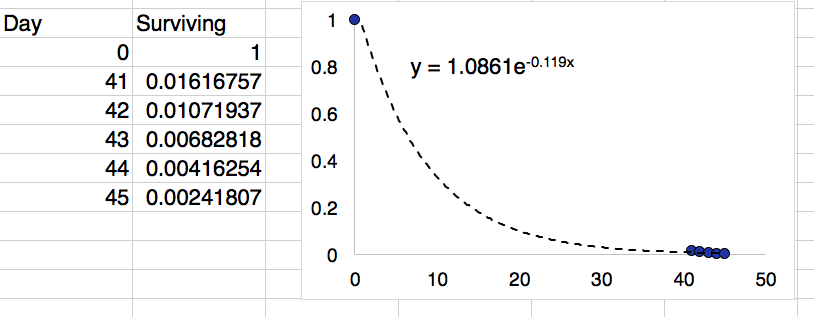
***

***27ºC Block 1***

***
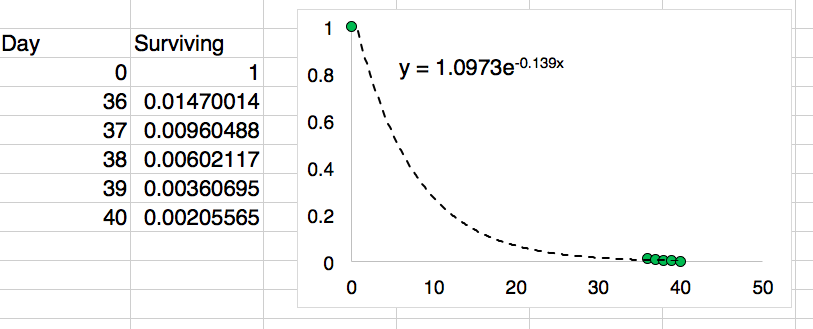
***

***27ºC Block 2***

***
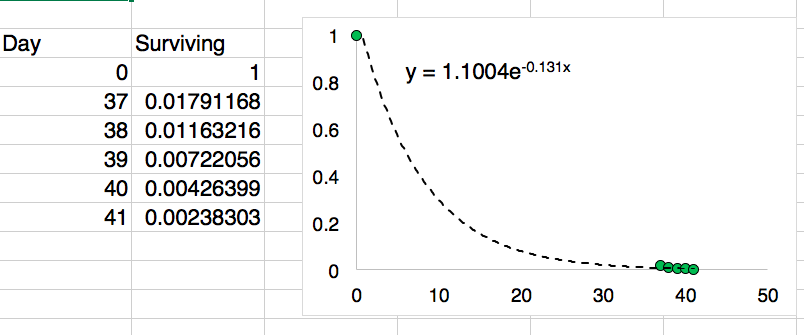
***

***30ºC Block 1***

***
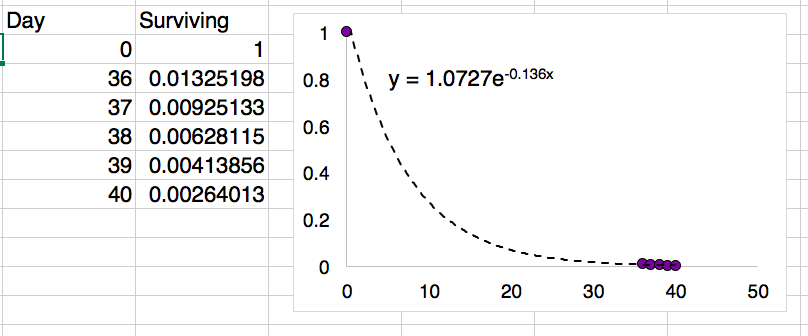
***

***30ºC Block 2***

***
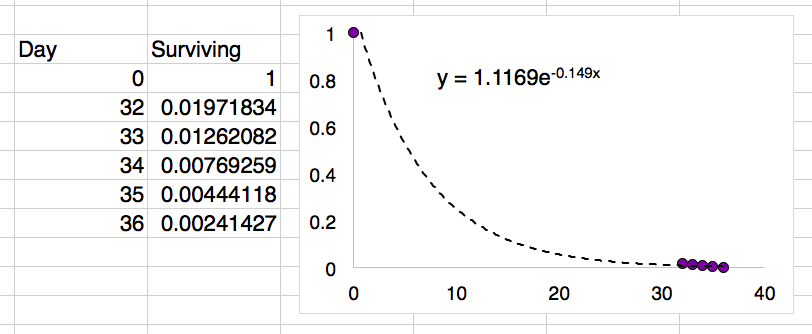
***

***32ºC Block 1***

***
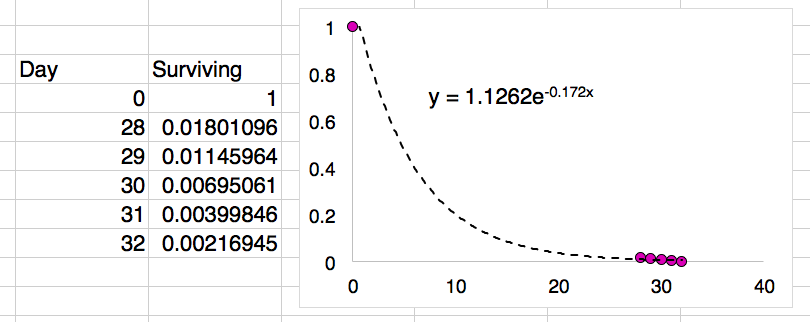
***

***32ºC Block 2***

***
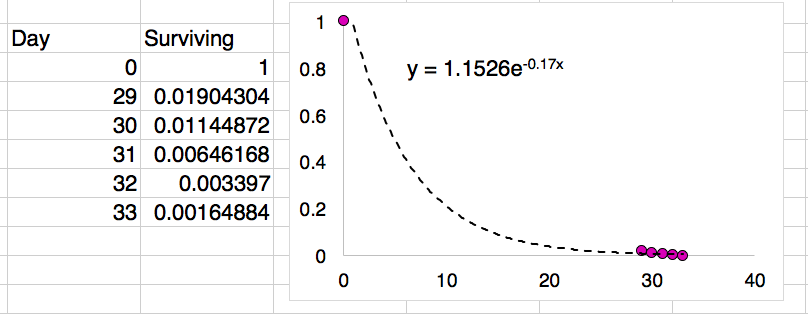
***

***34ºC Block 1***

***
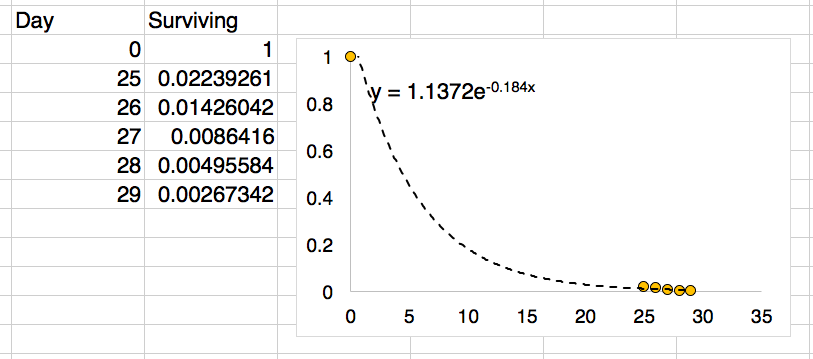
***

***34ºC Block 2***

***
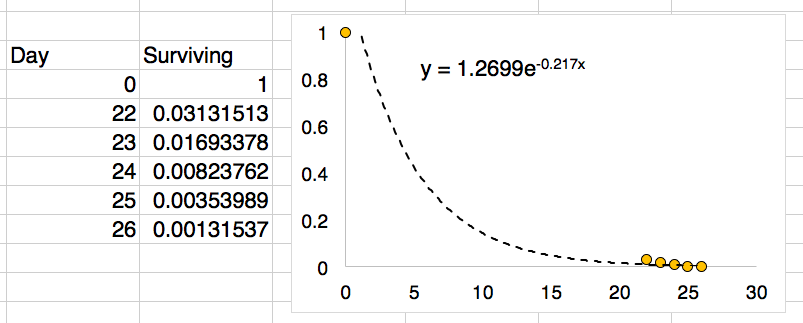
***
